# Computer-Vision-Enabled Worker Video Analysis for Motion Amount Quantification

**DOI:** 10.3390/s26165153

**Published:** 2026-08-14

**Authors:** Hari Iyer, Neel Macwan, Shenghan Guo, Heejin Jeong

**Affiliations:** 1 The Polytechnic School, Ira A. Fulton Schools of Engineering, Arizona State University, Mesa, AZ 85212, USA; hniyer@asu.edu; 2 School of Manufacturing Systems and Networks, Ira A. Fulton Schools of Engineering, Arizona State University, Mesa, AZ 85212, USA; nhmacwan@asu.edu (N.M.); shenghan.guo@asu.edu (S.G.)

**Keywords:** computer vision, Hotelling’s *T*^2^, in situ videos, joint motion amount, posture estimation

## Abstract

The performance of physical workers is significantly influenced by the extent and quality of their motions. However, accurately measuring and assessing these motions remains a challenge due to the limitations in conventional instrumentation; wearable sensors require calibration and restrict mobility, while marker-based motion capture systems are costly and impractical for field deployment. Recent advancements have enabled in situ video analysis for the real-time observation of worker behaviors. To address these measurement constraints, this paper introduces a novel framework for tracking and quantifying upper and lower limb motions, issuing alerts when critical thresholds are reached. Using joint position data from posture estimation, the framework employs Hotelling’s $T^{2}$ statistic to quantify and monitor motion amounts. A significant positive correlation was noted between motion warnings and the overall NASA Task Load Index (TLX) workload rating (*r* = 0.218, *p* < 0.005). A supervised Random Forest model trained on the collected motion data was benchmarked across multiple datasets, including the in-house assembly dataset, G-AI-HMS, UCF Sports Action, UCF50, and PE-USGC. The proposed framework identified motion anomaly patterns with a maximum accuracy of 94% on the in-house assembly dataset, while performance varied across the external benchmark datasets.

## 1. Introduction

Worker motion analysis involves studying human movement within workplace environments. Sustained awkward postures and muscular overexertion often lead to work-related injuries [1]. The primary motivation for worker motion analysis is to improve workplace safety. By monitoring worker movements, safety professionals and ergonomists can detect potential risks, such as injuries caused by repetitive muscular strain and awkward postures [2,3]. Implementing safety measures and ergonomic improvements can help reduce the risk of injury.

Analyzing task execution in labor-intensive fields like manufacturing, construction, and agriculture can uncover redundant or wasteful movements. Gouett [4] analyzed activity for continuous productivity improvement in construction and found that applying this process continually to a construction site can significantly improve direct-work rates throughout a project’s duration. This optimization not only increases productivity but also reduces unnecessary strain on workers. Worker motion analysis is closely associated with ergonomics [5], which is the science of designing workplace tools and tasks to fit the worker. For example, Golabchi et al. [6] developed a programmed simulation of biomechanical systems for the design analysis of workplace ergonomics. This approach lowers ergonomic risks by detecting uncomfortable postures in virtual models. Understanding how workers interact with products and equipment allows designers to create tools that are easier and more comfortable to use.

Worker motion analysis supports training and skill development. Organizations can develop motion simulation-based programs to train new employees efficiently [7]. Motion analysis can identify areas where workers may require additional support or training. A critical aspect of worker motion analysis is human data collection capability. Recently, with the advances in sensing and artificial intelligence (AI) technologies, real-time sensing has enabled task-based data collection [8]. For example, the posture- and position-based workers’ safety risk evaluation framework developed by Chen et al. [9] demonstrates that an automated evaluation of safety risk extent, combining spatial and postural factors, is almost 85% accurate, outperforming the approximately 80% accuracy of single-feature-driven risk examination using location and 55% accuracy when using posture. This proactive approach mitigates the risk of musculoskeletal injuries. According to Ryu et al. [10], motion data can be collected automatically to assess worker posture across experience levels. This postural analysis identifies areas where workers need training to improve their performance levels.

Current techniques for processing and analyzing human motion data are inefficient, making the monitoring and evaluation of joint movements challenging. Moran and Wallace [11] reviewed studies involving vertical jumps and noted findings that were contradictory, suggesting inconsistent levels of eccentric loading and range of joint motion as potential causes. Video-based frame analysis is an improvement over traditional methods for studying human motion. For the real-time processing of image frames, computer vision (CV) techniques use pose estimation and machine learning to extract actionable information [12]. Mehrizi et al. [13] estimated three-dimensional posture and joint kinematics during symmetrical lifting tasks using computer-vision-based motion capture and compared the results with those obtained from a marker-based system. Their results showed close agreement between the computer-vision-based and marker-based methods. Seo et al. [14] developed a CV model for posture classification to perform automated ergonomic assessment, achieving over 90% accuracy. Various methods are selected based on task and industry demands. In situ videos provide real-time information about ergonomic behavior, but scalability in posture extraction and processing remains challenging. These studies collectively demonstrate the growing feasibility of applying computer vision to ergonomic analysis while also revealing key limitations that motivate this paper.

**Figure 1:**
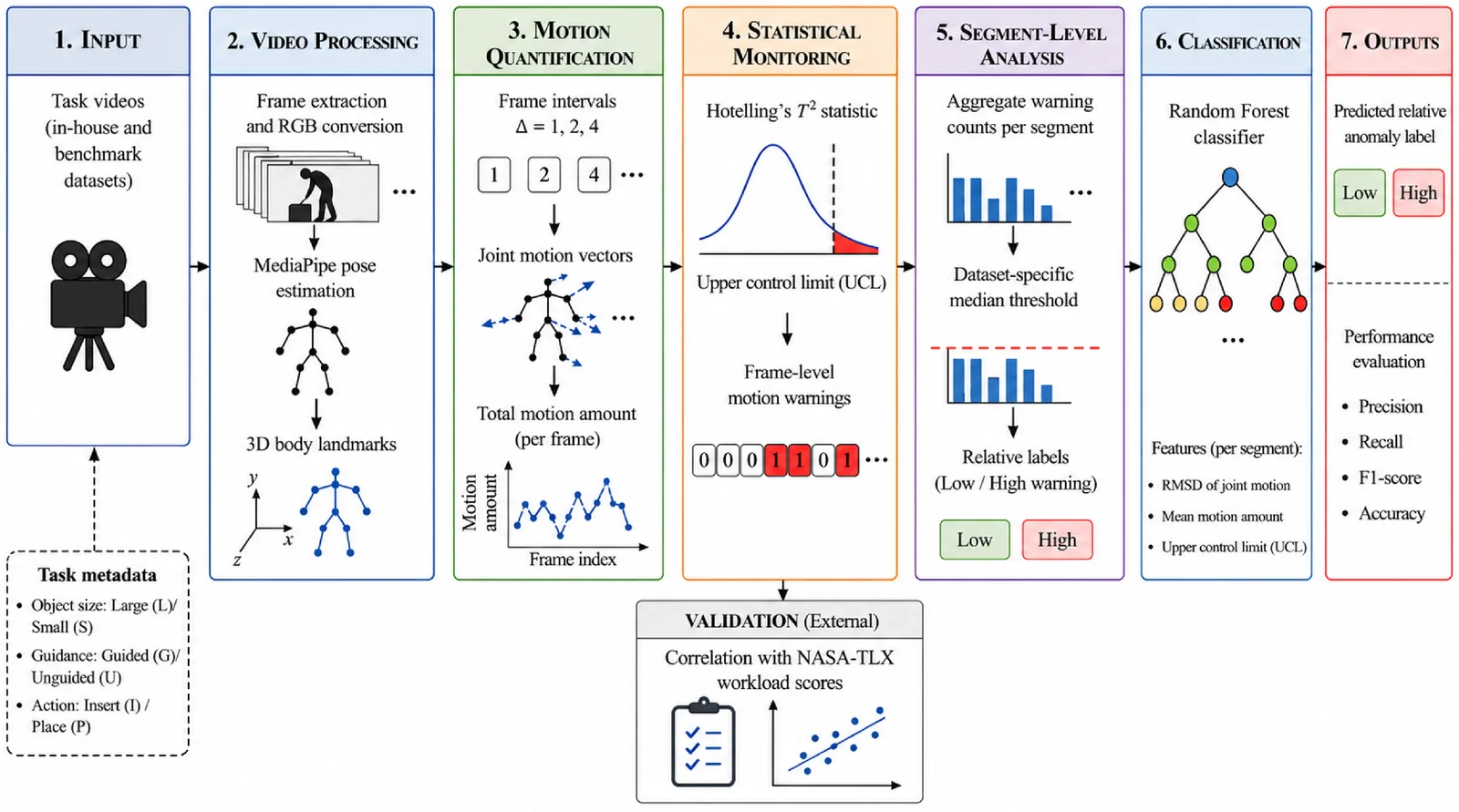
Architecture pipelines of posture estimation and worker motion analysis from the videos captured of assembly tasks.

To address these limitations, we propose an automated framework (see Figure 1). The framework uses CV techniques to extract body features from task videos through posture estimation [15,16]. MediaPipe identifies 33 body landmarks; we focused on key landmark points for motion quantification [17]. Hotelling’s $T^{2}$ control charts were built to flag significant “anomalies” [18]. Correlation analysis was performed between the detected anomalies and ground-truth motion information to evaluate motion-detection validity. We further implement a Random Forest classification model trained on motion statistics and warnings to assess the proposed framework across diverse task domains. The proposed framework can be implemented in real time to quantify and monitor worker motions. The notation used throughout the proposed motion quantification and risk detection framework is summarized in Table 1. This paper contributes to the field of instrumentation and measurement by transforming in situ video data into quantifiable joint motion metrics using posture estimation pipelines and multivariate statistical monitoring. The proposed framework uses computer vision to collect motion data, quantify joint movement, and apply Hotelling’s $T^{2}$ method to detect motion anomalies for ergonomic assessment and task monitoring. There are six key contributions of this work:

1. We present a video-based framework for the motion quantification and detection of motion anomalies associated with potential ergonomic risk using a multivariate monitoring of body-landmark trajectories.
2. We implement a Hotelling’s $T^{2}$-based control-chart methodology to detect deviations in motion patterns and generate statistical motion-warning thresholds.
3. We conduct an ablation study comparing full-body and partial landmark subsets (hands, upper body, lower body) across five benchmark datasets.
4. We introduce an adaptive thresholding mechanism using dataset-specific medians to improve the classification of motion warnings.
5. We demonstrate the effectiveness of our method through comparative classification performance, highlighting robustness across varied physical task domains and dataset types.
6. We incorporate multiple benchmark datasets, including G-AI-HMS [19], UCF Sports Action [20,21], UCF50 [22], and PE-USGC [12].

The remainder of this paper is organized as follows. Section 2 reviews state-of-the-art literature on worker behavioral analysis and data science methods in this field. Section 3 describes the experimental designs and data collection process. Section 4 details the technical aspects of the proposed framework. Section 5 presents the results, and Section 6 discusses the findings. Finally, Section 7 concludes the paper with a summary and future directions.

## 2. Literature Review

### 2.1. Hierarchical Approaches to Learning Human Behavior

Recognizing human activities in videos remains challenging because both spatial and temporal movement patterns must be interpreted. Early studies addressed this problem through hierarchical and probabilistic modeling. Robertson and Reid [23] proposed a hierarchical approach that combined trajectory information with local motion descriptors. However, the method required a large database and accurate motion evaluation, which limited its practical applicability. Similarly, Bregler [24] used expectation-maximization clustering, dynamical systems, and hidden Markov models to learn human dynamics from videos. Building on these model-based approaches, Duchenne et al. [25] introduced a weakly supervised learning method that extracted video features using scripts, clustered them according to feature similarity, and trained a temporal action detector on the resulting groups. In a related multimodal approach, Han and Bhanu [26] used a hierarchical genetic algorithm to establish correspondence between color and thermal images. Hoai et al. [27] further integrated video segmentation and action recognition using a multi-class support vector machine and dynamic programming. Collectively, these studies demonstrate the value of hierarchical and temporal modeling for human-behavior analysis, although many require complex training procedures, substantial data, or detailed annotations. Rapid human movement also remains a challenge for reliable video analysis. In contrast, this paper uses computer vision (CV) to extract body landmarks and treats worker-motion analysis as a pose-estimation and statistical-monitoring problem.

### 2.2. CV Approaches for Human Task Video Analysis

More recent research has shifted toward deep-learning architectures that jointly capture spatial and temporal information from videos. These approaches have achieved strong performance in human-action recognition and behavior understanding [28,29,30]. For example, Ji et al. [31] used a three-dimensional convolutional neural network to extract spatial and temporal features, although the method involved substantial computational cost. To reduce temporal redundancy, Wang et al. [32] introduced a temporal segment network that focused on selected video segments. Similarly, MVFNet [33] combined multi-view three-dimensional signals to capture video dynamics efficiently.

In parallel, pose-estimation studies have focused more directly on representing body posture and joint trajectories. Charles et al. [34] used frame-level annotations for posture detection, whereas Kanazawa et al. [35] incorporated a temporal encoder for three-dimensional human-motion analysis. These developments subsequently enabled real-time pose-estimation models, which typically use deep-learning architectures to detect body landmarks [15,36]. Beyond action recognition, Yu et al. [37] and Yu et al. [38] combined CV with insole pressure sensing for ergonomic workload assessment. However, these studies primarily address posture estimation, activity recognition, or workload assessment rather than continuous motion quantification combined with multivariate statistical warning generation. In contrast, this paper focuses on quantifying motion levels across comparable action distributions. Accordingly, MediaPipe’s pose estimator [17], which is based on BlazePose [15], was selected as the landmark-extraction component of the proposed motion-monitoring framework.

Although BlazePose was introduced in 2019, it remains suitable for tracking ergonomic movements because of its real-time responsiveness, low computational requirements, and reported accuracy across different movement types. Recent evaluations provide additional support for its use. Latreche et al. [39] reported high agreement for shoulder-joint motion with intra-class correlation coefficients exceeding 0.80 and angular discrepancies as small as −0.6 degrees compared with standard goniometric and inclinometer measurements. Likewise, Hamilton et al. [40] found that MediaPipe provided smooth joint-trajectory tracking, coefficients of variation below 10%, and strong intra-class consistency with Qualisys three-dimensional motion capture for elbow and knee flexion-extension measurements. Collectively, these findings support MediaPipe as a practical approach for quantifying movement in workplace contexts.

### 2.3. Research Gaps in Worker Motion Analysis

Worker motion analysis is important for workplace safety, productivity, and ergonomic design. Prior studies have demonstrated the value of worker-centered workspace design and ergonomic assessment [5,6], while workflow analysis has shown the potential to improve productivity and reduce unnecessary motion [4]. However, challenges remain in evaluating worker performance consistently across tasks and environments, highlighting the need for more robust and generalizable methods [11].

Wearable-sensor approaches using inertial measurement units (IMUs), surface electromyography (EMG), and accelerometers provide continuous kinematic and physiological information and can remain effective during visual occlusion. Their value for physical workload and ergonomic assessment has been demonstrated in systematic reviews and workplace studies [41]. Embedded IMU–EMG systems have been validated for lifting analysis [42], while multi-sensor IMU systems have been used to estimate trunk flexion, lifting duration, and load position [43]. Surface EMG combined with machine learning has also been applied to ergonomic risk prediction during manual material handling [44], and wearable IMU systems have supported posture and motion recognition in construction settings [45]. Despite these advantages, wearable methods often require multiple body-mounted sensors, calibration, and careful placement, which can limit scalability and practical deployment in industrial environments.

Vision-based methods offer a non-intrusive alternative, but important limitations remain. Existing approaches commonly emphasize posture estimation, activity recognition, or isolated ergonomic assessments rather than the continuous quantification of multijoint motion over complete task sequences. Their performance may also depend on camera viewpoint, visibility, task-specific models, and controlled recording conditions. Accordingly, there remains a need for non-intrusive systems that can detect motion deviations in real-world work conditions and support preliminary ergonomic screening [9]. Although AI-assisted worker training has also been identified as a promising application [10], a unified framework that combines pose estimation, interpretable motion quantification, multivariate statistical monitoring, and task-level classification remains limited.

The proposed framework addresses this gap by integrating video-based pose estimation, motion-amount quantification, Hotelling’s $T^{2}$ monitoring, and motion-anomaly classification within a single workflow. Unlike prior vision-based ergonomic studies that primarily focus on posture estimation or activity recognition, the proposed method evaluates temporal changes in multijoint motion and generates statistical alerts for motion patterns associated with potential ergonomic risk. The contribution of this paper therefore lies in the end-to-end integration of non-intrusive motion measurement, statistical process monitoring, and learning-based screening rather than in the development of any individual algorithmic component.

## 3. Collected and Benchmark Data Description

### 3.1. Experiment Data Collected

**Figure 2:**
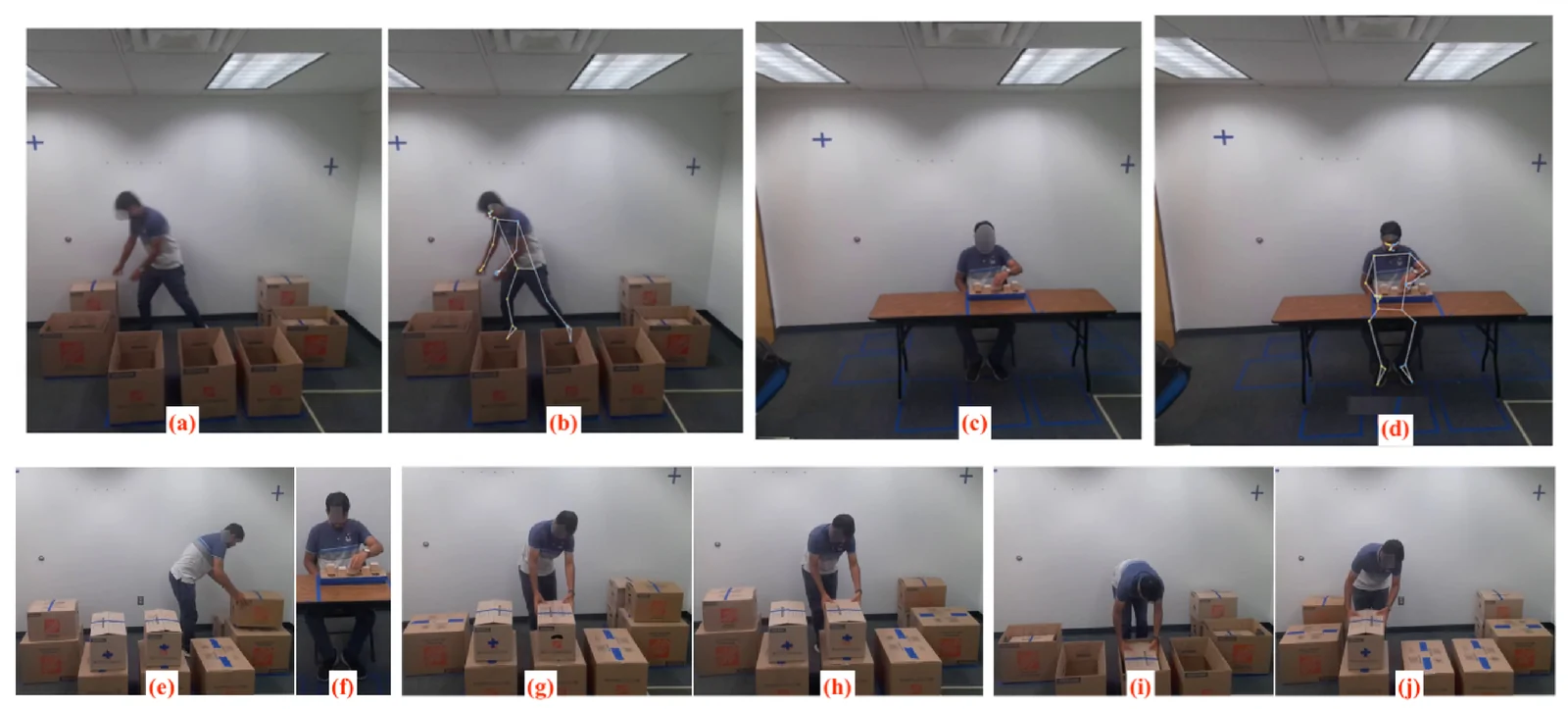
(**a**) Standing assembly task with feet obscured. (**b**) Posture estimation with MediaPipe to detect obscured feet. (**c**) Sitting assembly task with trunk and lower limbs obscured. (**d**) Posture estimation with MediaPipe to detect obscured trunk and lower limbs. (**e**) L task, where the participant moves big cardboard boxes. (**f**) S task, where the participant moves small wooden cubes. (**g**) G task, where the participant follows a guided order as seen in the specific placement of the boxes. (**h**) U task, where the participant follows an unguided sequence as seen in the serial (random) placement of the boxes. (**i**) I task, where the participant inserts the box or cube inside the cavity. (**j**) P task, where the participant places the box or cube on the platform. The tasks involve the guided or unguided insertion or placement of boxes and cubes.

The experimental design consisted of three components, resulting in eight tasks per participant (2 × 2 × 2). Participants engaged in tasks with objects of different sizes: large boxes (L) or small wooden cubes (S) (see Figure 2). They followed either guided (G) with a numbering/alphabet system or unguided (U) with random placements. Tasks involved inserting (I) objects into a cavity or placing (P) them on top of a surface. The codes (L, S, G, U, I, and P) were used in combination (e.g., SGI, LUP; see Figure 2) to label videos for each variable combination. Video recordings from two healthy males (age 27 ± 1.41 years; height 1.74 ± 0.014 m; arm length 0.70 ± 0.014 m) were collected at 30 frames per second (totaling approximately 5000 frames per participant). The Institutional Review Board (IRB) at Arizona State University approved the experimental protocol (STUDY00016442). Before participating in the experiment, participants read and signed an IRB-approved informed written consent form.

The experiment included guided and unguided techniques to test how explicit guidance affects participants’ performance. Guided tasks required structured, sequential instructions based on a numbering system, while unguided tasks involved random, self-directed actions. The workspace was kept free of obstacles to isolate the effects of object size, technique, and action type. Video cameras were positioned at one frontal 0-degree angle and two side angles at 45 degrees to capture upper and lower body motions. Video data were recorded using three devices: a Canon VIXIA 4K camcorder, a consumer-grade 4K camcorder, and a Samsung Galaxy Android smartphone. The workspace measured approximately 4.2 m × 3.9 m with a height of 2.4 m, providing ample space for participants to perform tasks without obstruction. The total floor area was approximately 16.4 m^2^. Multiple camera angles minimized blind spots and allowed for a detailed analysis of task strategies.

Videos were recorded at 30 frames per second using a Canon VIXIA 4K camcorder, a consumer-grade 4K camcorder, and a Samsung Galaxy Android smartphone. All experiments and data processing were conducted on a workstation equipped with an Intel Core i9 processor, 64 GB RAM, and an NVIDIA RTX A4000 GPU. The objective of this paper was to evaluate the proposed motion quantification framework rather than benchmark computational performance. Accordingly, processing time, end-to-end latency, and real-time throughput were not systematically evaluated and will be investigated in future work.

Recorded videos were manually labeled by task (e.g., SGI, LUP) based on the experimental protocol and organized with metadata (participant ID, trial number, camera angle). MediaPipe was applied to extract 3D landmarks from each frame for motion quantification and statistical analysis. Because the data were collected from only two participants with similar anthropometric characteristics, the experimental design does not support claims of inter-subject generalization, and all results should be interpreted as task-domain and dataset-level findings.

### 3.2. Benchmark Datasets

To assess how well our framework generalizes across tasks, we used multiple publicly available benchmark datasets covering diverse motion patterns and task types. The G-AI-HMS dataset [19] includes synthetic but high-fidelity human motions generated using a generative AI pipeline and validated through pose estimation. These tasks range from upper-limb activities to whole-body motions. The UCF Sports Action dataset [20,21] comprises authentic sports footage with full-body movements recorded in uncontrolled settings, which presents a challenging benchmark for motion-based classification. The UCF50 dataset [22] includes a wide variety of human actions sourced from online videos, which are representative of high inter-class variability, background noise, and occlusions. The PE-USGC dataset [12] presents near-duplicate repetitive culinary tasks. These datasets were selected to assess performance across structured, semi-controlled, and dynamic physical environments. For the in-house experimental dataset, we also collected NASA TLX subjective ratings [46], which served as a perceptual validation benchmark for the motion-based risk warnings.

## 4. Motion Amount Quantification and Data Analysis Methodology

### 4.1. Body Landmark Extraction with CV Tools

We used MediaPipe [17] for a CV analysis of landmark extraction from video frames. This process involves identifying key landmark points. These landmarks represent upper and lower limbs joints. Motion is calculated from positional changes between frames [47] for frame steps (0, 2, 4). We treat the data as a continuous multivariate stream drawn from a single distribution. Feature vectors per frame capture spatial landmark data. Data smoothing was intentionally omitted to preserve the raw temporal characteristics of the recorded motion and to reflect real-time deployment conditions. Consequently, frame-to-frame landmark jitter may contribute to small fluctuations in the estimated motion vectors. However, the proposed framework evaluates motion over complete task sequences using multivariate statistical monitoring rather than isolated frame-level displacements, reducing the influence of occasional pose estimation noise. Nevertheless, landmark jitter remains a potential source of variability and should be considered when interpreting the results.

The landmark points selected for the S and L tasks (see Table 2) were used to analyze task-specific movements. For S tasks, involving precise hand movements with small cubes, landmark points such as the left and right elbows (Indexes 13, 14), wrists (15, 16), and various finger joints including pinkies (17, 18), indexes (19, 20), and thumbs (21, 22) were selected to capture fine motor movements. For L tasks with larger boxes, landmark points like left and right shoulders (11, 12) and knees (25, 26) were selected to help analyze larger, exertive movements. By targeting these specific landmark points, researchers and ergonomists can create interventions to reduce workplace injuries and improve efficiency, for task-specific demands.

To calculate the motion amount, we first define the position vector for each landmark *i* in frame *k* as shown in Equation (1).

(1) $$v_{i,k}=\left(x_{i,k},y_{i,k},z_{i,k}\right)$$

where $v_{i,k}$ represents the 3D position vector of landmark *i* at frame *k* with $x_{i,k}$, $y_{i,k}$, and $z_{i,k}$ as its Cartesian coordinates. All landmark coordinates were analyzed in the normalized coordinate space returned by MediaPipe. No camera calibration or conversion to physical units was performed. The MediaPipe coordinates were normalized relative to the image frame, but no additional normalization with respect to participant height, limb length, or any other anthropometric reference was performed. Consequently, the estimated displacement values may be influenced by participant body size, camera-to-participant distance, camera viewpoint, image framing, and perspective. Within the controlled Assembly dataset, these effects were reduced through consistent camera placement and similar participant anthropometric characteristics. However, differences in scale and viewpoint may contribute to distribution shifts when the framework is applied to independently collected datasets. For this reason, the cross-dataset results should be interpreted as an evaluation of framework transfer under heterogeneous recording conditions rather than as a direct comparison of absolute physical movement magnitudes. Future implementations may incorporate body-scale normalization, such as normalization by estimated standing height, shoulder width, or torso length, together with camera calibration or view-invariant coordinate transformation. Therefore, the derived motion amount, RMSD, mean motion, and motion standard deviation are dimensionless.

The motion $m_{i,k}$ for each landmark is then defined as the vector difference between consecutive frames. Motion was evaluated using three temporal frame intervals referred to as Step 0, Step 2, and Step 4. Step 0 represents motion computed between consecutive frames (i.e., frame *k* and frame k−1), while Step 2 and Step 4 represent motion computed between frames separated by two and four previous frames, respectively. These frame intervals were selected to assess the robustness of motion quantification across different effective temporal resolutions. Step 0 retains consecutive-frame information, whereas Steps 2 and 4 approximate lower effective sampling rates by increasing the temporal separation between compared frames, reflecting potential real-world deployments with reduced frame availability.

(2) $$m_{i,k}=v_{i,k}-v_{i,k-\Delta }$$

where $m_{i,k}$ is the motion vector of landmark *i* between frame *k* and the reference frame k−Δ, where Δ=1 for Step 0, Δ=2 for Step 2, and Δ=4 for Step 4. The concatenated motion vector is defined as $m_{k}=\left[m_{1,k},m_{2,k},\ldots ,m_{p,k}\right]$.

The total motion amount $M_{k}$ in frame *k* is then computed as the sum of magnitudes of each of the *p* landmarks’ motion vector calculated in Equation (2).

(3) $$M_{k}=\sum_{i=1}^{p}∥m_{i,k}∥$$

It is important to note that the scalar motion amount $M_{k}$ defined in Equation (3) is used as a descriptive and interpretable summary of overall movement intensity at frame *k*. This scalar aggregation is not used for multivariate statistical monitoring. Instead, joint-wise motion vectors are retained in their original multivariate form for subsequent covariance-based analysis. This calculation quantifies the overall motion $M_{k}$ in each frame by summing the magnitudes of all *p* landmark motion vectors, capturing the total activity of all landmarks in the frame sequence.

### 4.2. Motion Amount Monitoring and Warning

In this paper, we propose using a control chart for the systematic monitoring of worker demand statistics. Specifically, the Hotelling’s $T^{2}$ control chart [18,48] tracks key features. An upper control limit (UCL) flags outliers as potential warning levels. We use the Hotelling’s $T^{2}$ statistic for warnings based on the joint motion. Williams et al. [49] showed that using $T^{2}$ statistics derived from successive-difference estimators improves UCL accuracy for individual observations during a Phase I analysis. This statistic monitors the multivariate distribution of the selected features across video frames. Accordingly, scalar motion metrics and multivariate monitoring serve complementary roles in the proposed framework, with the former supporting interpretability and learning-based classification, and the latter enabling statistically rigorous anomaly detection.

### 4.3. Monitoring Statistic

While scalar motion summaries are useful for descriptive analysis, statistical monitoring in this paper is performed using the full multivariate joint motion representation. Specifically, Hotelling’s $T^{2}$ statistic is computed from the concatenated joint motion vector $m_{k}=\left[m_{1,k},m_{2,k},\ldots ,m_{p,k}\right]\in R^{3p}$, preserving the inter-joint coordination and covariance structure. This formulation ensures that the statistical monitoring framework operates on a multivariate random vector consistent with the assumptions of Hotelling’s $T^{2}$ control charts. Accordingly, we define the matrix *V* representing the positions of selected landmarks in 3D space over *K* frames, where each frame contains Cartesian coordinates for the selected landmark indices, as shown in Equation (4).

(4) $$V={\left[\begin{array}{cccc} v_{13,1} & v_{14,1} & \cdots & v_{22,1}\\ v_{13,2} & v_{14,2} & \cdots & v_{22,2}\\ \vdots & \vdots & \ddots & \vdots \\ v_{13,K} & v_{14,K} & \cdots & v_{22,K} \end{array}\right]}_{K\times 3p}$$

where each entry $v_{i,k}=\left(x_{i,k},y_{i,k},z_{i,k}\right)$ denotes the Cartesian coordinates of landmark *i* in frame *k*. The matrix dimensions are K×3p with *K* representing the number of frames and *p* representing the total number of landmarks points (e.g., 13 to 22 in this case).

The sample covariance matrix is defined as

(5) $$S=\frac{1}{K-1}\sum_{i=1}^{K}\left(m_{k}-\bar{m}\right){\left(m_{k}-\bar{m}\right)}^{T}$$

Although $S\in R^{3p\times 3p}$ is high-dimensional, its estimation in this paper remains well conditioned due to the large sample regime and task-specific landmark selection. Specifically, only a subset of landmarks is used for each task (Table 2), resulting in modest values of *p*, while the number of frames *K* per video segment is substantially larger than 3p in all experiments. Empirically, the resulting covariance matrices were full rank and invertible for the stable computation of the Hotelling’s $T^{2}$ statistic without additional regularization. This setting is consistent with Phase I multivariate statistical process monitoring, where covariance estimation is performed using a sufficiently large historical sample.

For example, the hand movements in frame *k* use landmarks from V like $v_{13,k}=\left(x_{13,k},y_{13,k},z_{13,k}\right),v_{14,k}=\left(x_{14,k},y_{14,k},z_{14,k}\right),\ldots$ to calculate aggregate motion vector $m_{k}$.

Based on Equation (5), the monitoring statistic for frame *k* is calculated as

(6) $$t_{k}=\left(m_{k}-\bar{m}\right)S^{-1}{\left(m_{k}-\bar{m}\right)}^{T}$$

where $t_{k}$ is the monitoring statistic value for the frame *k* assessing the extent to which the worker’s motion deviates from the reference distribution relative to the distribution.

Calculating a control value based on Equation (6) for each instance in frames gives us the following vector:

(7) $$T=\left[\begin{array}{c} t_{1}\\ t_{2}\\ \vdots \\ t_{K} \end{array}\right]$$

It is unnecessary to calculate the mean of the mean vectors since the data are not divided into subgroups. Thus, our control values are derived as above in Equation (7). The upper and lower control limits, UCL and LCL, are given in Equations (8) and (9), respectively [18,50]. *F* is a critical value from the F-distribution (shown in Appendix V of [18]) at a significance level α with the degrees of freedom 3p (numerator) and K−3p (denominator).

(8) $$\mathrm{UCL}=\frac{3p(K+1)(K-1)}{K^{2}-3Kp}F_{1-\alpha /2,3p,K-3p}$$

(9) LCL=0

Because this paper focuses on methodological validation, formal multivariate normality tests were not performed. The Hotelling’s T^2^ control limits were computed using the conventional large-sample F-distribution approximation, which is appropriate because the number of observations substantially exceeded the feature dimensionality.

### 4.4. Control Limits

The Hotelling’s $T^{2}$ statistic is non-negative by definition, and therefore the LCL is set to zero as a mathematical bound rather than an interpretive threshold [18]. In this paper, the LCL does not convey information about motion quality or performance. Instead, only exceedances of the UCL, derived from the *F*-distribution, are used to identify statistically significant deviations in joint motion patterns. Accordingly, frames with $T^{2}$ values near zero simply indicate proximity to the estimated mean motion profile and are not interpreted as indicators of improved or degraded motion quality.

The control limits in this paper were computed using the large-sample *F*-distribution approximation for Phase I Hotelling’s $T^{2}$ control charts. Formal multivariate normality testing was not performed because the primary objective was methodological validation of the proposed monitoring framework rather than statistical inference. This approximation is appropriate for this paper because the number of analyzed frames (*K*) substantially exceeds the dimensionality of the multivariate motion vector (3p), enabling stable estimation of the covariance matrix and control limits.

### 4.5. Correlation Analysis to Validate Significant Motions

To assess motion variation, we compute the Root Mean Square Deviation (RMSD) [51], focusing on tasks involving smaller objects (S tasks), which typically exhibit intricate, frequent movements. The RMSD is calculated using Equation (10).

(10) $${\mathrm{RMSD}}_{\mathrm{task}}=\sqrt{\frac{1}{K}\sum_{k=1}^{K}{\left(M_{k}-\bar{M}\right)}^{2}}$$

where $\bar{M}$ is the mean motion amount across all frames, which is calculated similar to Equation (3). The full pipeline for motion quantification and adaptive motion-anomaly classification is summarized in Algorithm 1.

Although both RMSD and Hotelling’s $T^{2}$ relate to deviations from a nominal motion profile, they capture fundamentally different aspects of worker motion. ${\mathrm{RMSD}}_{task}$ is a scalar measure that summarizes the temporal variability of the overall motion magnitude $M_{k}$ across an entire task segment, making it suitable for workload correlation analysis and learning-based classification. However, Hotelling’s $T^{2}$ is a frame-level multivariate statistic that captures instantaneous extremeness in joint coordination relative to the covariance structure. $T^{2}$ is used for statistical process monitoring and warning generation, while RMSD provides a complementary aggregate descriptor of motion variability over time.
**Algorithm 1** Motion Quantification and Adaptive Motion-Anomaly Classification Pipeline  1:**Input:** Raw task video directory V, list of landmark sets L, step sizes S  2:**Output:** Labeled dataset with warnings, classifier performance metrics  3:**Stage 1: Landmark Extraction via MediaPipe**  4:**for** each video folder v∈V **do**  5:    **for** each frame f∈v **do**  6:        Read frame and convert to RGB  7:        Extract 3D landmarks using MediaPipe: $v_{i,k}=\left(x_{i,k},y_{i,k},z_{i,k}\right)$  8:    **end for**  9:    Save per-frame landmark matrix to CSV10:**end for**11:**Stage 2: Motion Quantification Across Frames**12:**for** each CSV file **do**13:    **for** each step size s∈S **do**14:        **for** each landmark set ℓ∈L **do**15:            Compute motion vectors: $m_{i,k}=v_{i,k}-v_{i,k-s}$16:            Compute total motion: $M_{k}=\sum_{i=1}^{p}∥m_{i,k}∥$17:            Compute Hotelling’s $T^{2}$ statistic for each frame: $t_{k}$ {Equation (6)}18:            Estimate control limit: UCL from F-distribution {Equation (8)}19:            Count warnings: $w=\sum_{k}\left[t_{k}>\mathrm{UCL}\right]$20:            Calculate RMSD of motion: $\mathrm{RMSD}=\sqrt{\frac{1}{K}\sum_{k}{\left(M_{k}-\bar{M}\right)}^{2}}$21:            Append metrics to results table22:        **end for**23:    **end for**24:**end for**25:**Stage 3: Adaptive Warning Labeling**26:**for** each dataset *D* **do**27:    Compute median warning value: $\theta_{D}=\mathrm{median}\left(w_{D}\right)$28:    Label instances: $y_{k}=⊮\left(w_{k}>\theta_{D}\right)$29:**end for**30:**Stage 4: Classifier Training and Evaluation**31:Select source dataset $D_{\mathrm{train}}=\mathrm{Assembly}$32:Train Random Forest on $D_{\mathrm{train}}$: features = [RMSD, mean motion, UCL]33:**for** each dataset $D_{\mathrm{test}}$ **do**34:    Predict warning labels using trained model35:    Compute classification metrics: precision, recall, accuracy, F136:**end for**37:**return** Labeled results, classification performance metrics

### 4.6. Random Forest-Based Classification of Motion-Anomaly Warnings

The objective of the classification stage is not to independently determine ergonomic risk but to efficiently approximate the motion anomaly patterns identified by the Hotelling’s $T^{2}$ monitoring framework using computationally inexpensive task-level motion features. Rather than performing frame-by-frame multivariate statistical analysis during inference, the classifier provides a coarse screening mechanism based on aggregate motion descriptors, enabling an efficient identification of video segments that may warrant further statistical evaluation. Accordingly, the classification model predicts relative motion anomaly levels derived from the proposed monitoring framework and should not be interpreted as providing independent ground-truth ergonomic risk assessment.

To assess whether motion-derived features can generalize motion-anomaly classification across tasks, we used a supervised learning pipeline with Random Forests. The objective was to predict whether a task video segment should be assigned a relatively high-warning label using motion statistics and control-chart outputs. Hotelling’s $T^{2}$ control chart is used in this framework to identify frame-level motion anomalies within individual videos. The resulting warning counts are then used only to construct segment-level binary labels via the adaptive thresholding rule in Equation (11). Importantly, the learning model is not trained on frame-level $T^{2}$ values or on the warning counts themselves; instead, it is trained on the task-level motion summary features derived from $M_{k}$ (RMSD, mean motion, and motion standard deviation), which characterize the overall motion intensity and variability within a segment. This distinction separates the monitoring stage, which generates weak segment-level labels from frame-level $T^{2}$ exceedances, from the classification stage, which uses aggregate motion features that exclude both frame-level $T^{2}$ values and warning counts.

#### 4.6.1. Label Assignment Using Adaptive Thresholding

Binary warning labels were generated using adaptive thresholding that dynamically adjusts to the baseline activity of each dataset. For each dataset *d*, a threshold $\tau_{d}$ was computed as the median number of motion-based warnings. Each video segment *i* was assigned a binary label $y_{i}$, indicating whether the number of warnings exceeded the threshold, as defined in Equation (11). This adaptive method balances each dataset without relying on a global threshold, which could be unsuitable due to variable baseline motion patterns across datasets.

For the benchmark datasets (G-AI-HMS, UCF Sports Action, UCF50, and PE-USGC), the original dataset action categories were not used as ergonomic risk labels. Instead, binary labels were generated independently for each dataset using the adaptive thresholding procedure described in Equation (11). Specifically, video segments with warning counts greater than the median number of warnings for that dataset were assigned a high-warning label, while the remaining segments were assigned a low-warning label. This approach provides a consistent relative labeling strategy across datasets with different motion characteristics.

(11) $$y_{i}=\left\{\begin{array}{cc} 1 & \mathrm{if}\;{\mathrm{warnings}}_{i}>\tau_{d}\\ 0 & \mathrm{otherwise} \end{array}\right.$$

The adaptive median threshold partitions video segments into relatively higher- and lower-warning groups within each dataset based on their motion characteristics. Consequently, the resulting binary labels represent relative anomaly levels within a dataset rather than absolute ergonomic risk levels, allowing consistent comparisons across datasets with different baseline motion distributions.

#### 4.6.2. Feature Construction

The classifier input included three features per video segment: RMSD of joint motion, mean motion amount, and the Hotelling-based UCL. These features capture variability in motion behavior and anomaly thresholds derived from multivariate monitoring. Each input sample $x_{i}\in R^{3}$ representing the task segments’ motion profile was constructed as shown in Equation (12).

(12) $$x_{i}=\left[{\mathrm{RMSD}}_{i},\mu_{\mathrm{motion},i},{\mathrm{UCL}}_{i}\right]$$

The three selected features were designed to capture complementary aspects of worker motion dynamics and anomaly detection. RMSD quantifies the overall variability of joint motion across frames, reflecting the magnitude of physical activity within a task segment. The mean motion amount represents the average movement intensity, while the RMSD captures the temporal variability in motion execution. The Hotelling-based UCL is a deterministic threshold parameter computed from Equation (8) using the segment length *K*, dimensionality 3p, and the chosen significance level α. It does not depend on the observed $T^{2}$ values within a segment and therefore does not encode the number of exceedances used to generate the label in Equation (11). In this feature set, UCL functions as a normalization parameter that accounts for segment-length and dimensionality effects when comparing segments across datasets and ablations. Preliminary ablation tests showed that incorporating all three features resulted in greater cross-dataset classification stability compared with single-feature models, supporting their inclusion in the final Random Forest classifier. Although the labels and input features originate from the same underlying motion sequences, the classifier does not receive the frame-level $T^{2}$ statistics or warning counts used to construct the labels. The classification results therefore measure how well aggregate motion features approximate the $T^{2}$-derived anomaly partition rather than providing an independent validation of ergonomic risk.

#### 4.6.3. Model Training and Inference

A Random Forest model $f_{\theta }$ was trained using the Assembly dataset. The model learns to associate the statistical features in $x_{i}$ with the warning labels $y_{i}$ derived from the $T^{2}$ control chart. For a new segment, the predicted label ${\hat{y}}_{i}$ is derived from the trained model, as shown in Equation (13). The output ${\hat{y}}_{i}\in \left\{0,1\right\}$ indicates whether the input motion segment is assigned a relatively low-warning label (0) or high-warning label (1).

(13) $${\hat{y}}_{i}=f_{\theta }\left(x_{i}\right)$$

The Random Forest classifier was implemented with 100 decision trees (n_estimators = 100) and balanced class weights to account for class imbalance. The Assembly dataset was randomly split into training (80%) and validation (20%) subsets using a fixed random seed (random_state = 42) to ensure reproducibility. Other hyperparameters, including the maximum tree depth and split criterion, were left at their default Scikit-learn settings.

The Random Forest aggregates predictions from an ensemble of *T* decision trees. Each tree $h_{j}$ makes its own prediction, and the final result is based on majority vote, as shown in Equation (14). This ensemble approach reduces overfitting and supports generalization across data domains.

(14) $$f_{\theta }\left(x\right)=\mathrm{mode}\left({\left\{h_{j}\left(x\right)\right\}}_{j=1}^{T}\right)$$

This classification layer provides segment-level decisions that extend the frame-level monitoring results and can be applied across tasks. Random Forest was selected because it provides robust performance on structured tabular features, requires minimal hyperparameter tuning, and performs well with relatively small training datasets. The objective of this paper was to evaluate the proposed motion quantification framework using a well-established classifier rather than to compare machine learning algorithms. Evaluating alternative classifiers remains an important direction for future work.

#### 4.6.4. Cross-Dataset Evaluation

After training on the Assembly dataset, the model was evaluated on all other datasets using the adaptive thresholding rule for labeling. Performance was quantified using standard classification metrics—precision, recall, F1-score, and accuracy.

Precision is the proportion of correctly identified positive instances out of all predicted positives, as shown in Equation (15).

(15) $$\mathrm{Precision}=\frac{TP}{TP+FP}$$

Recall, the proportion of correctly identified positives among all actual positives, is given in Equation (16).

(16) $$\mathrm{Recall}=\frac{TP}{TP+FN}$$

The F1-score is the harmonic mean of precision and recall, which is calculated using Equation (17).

(17) $$F1-\mathrm{score}=\frac{2\cdot \mathrm{Precision}\cdot \mathrm{Recall}}{\mathrm{Precision}+\mathrm{Recall}}$$

Finally, accuracy, the proportion of correctly classified instances over all instances, is shown in Equation (18).

(18) $$\mathrm{Accuracy}=\frac{TP+TN}{TP+TN+FP+FN}$$

Here, TP, FP, TN, and FN represent the number of true positives, false positives, true negatives, and false negatives, respectively. These metrics quantify classifier performance across domains and conditions of class imbalance.

## 5. Results

### 5.1. Assembly Task Analysis

The results show trends in motion amount, velocity, acceleration, landmark trajectories, and control-chart warnings. All standard deviation (SD) data in this manuscript refer to population SD. As shown in Table 3, the mean motion amount decreases at Steps 2 and 4, while velocity and its variability also decrease. Acceleration exhibits high initial variability (SD of 0.311 at Step 0), which decreases by Step 4. In Table 4, landmark point movement data indicate that smaller object tasks (S) have finer X/Y mean variation, whereas larger object tasks (L) show greater Z-axis variability, particularly for tasks LGI and LGP. Larger object tasks exhibit higher variability in landmark points and warnings, while motion amount and velocity data suggest greater movement stability. For S tasks, the RMSD of the aggregate joint motion amount was 0.072 compared with 0.035 for L tasks. Both values are dimensionless because the calculations were performed using normalized MediaPipe coordinates.

Table 5 shows the Pearson correlations between motion warnings generated by the framework and subjective workload ratings from the NASA-TLX subscales [52]. Statistically significant positive correlations were observed for Mental Demand (r = 0.192, *p* < 0.01), Physical Demand (r = 0.214, *p* < 0.005), Temporal Demand (r = 0.231, *p* < 0.005), and Effort (r = 0.155, *p* < 0.05), indicating that increased motion warnings tend to co-occur with higher perceived workload in these domains. Performance showed a weak, non-significant correlation (r = 0.109, *p* = 0.13), while Frustration was excluded due to incomplete data. The average correlation across subscales was 0.218 (*p* < 0.005), indicating a weak but statistically consistent association between objective motion warnings and subjective workload ratings.

**Figure 3:**
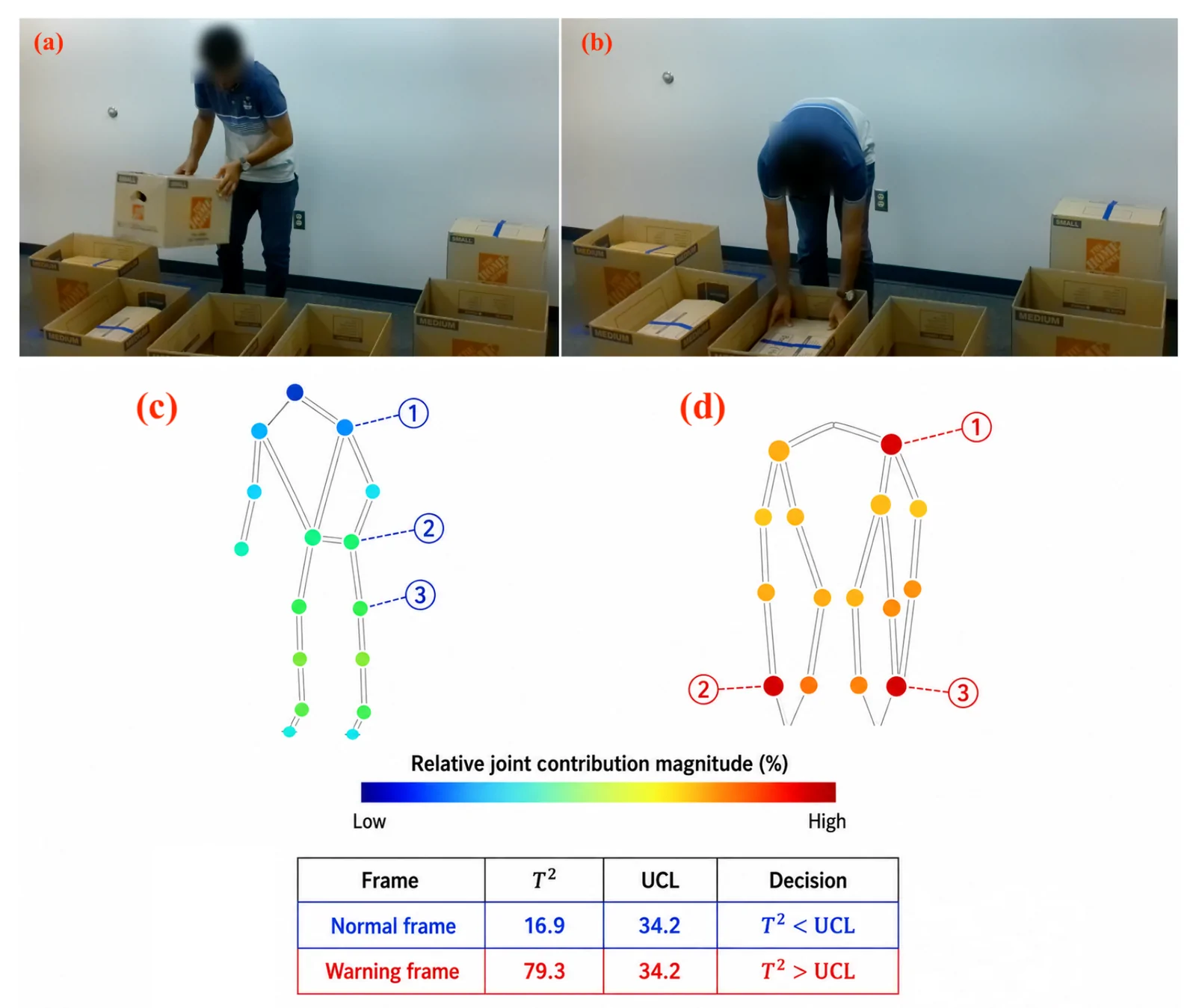
Representative examples and frame-level interpretations of the Hotelling’s $T^{2}$ monitoring results: (**a**) a normal frame with $T^{2}=16.9$, below the upper control limit of 34.2; (**b**) a warning frame with $T^{2}=79.3$, exceeding the upper control limit of 34.2; (**c**) a skeletal visualization of the normal frame; and (**d**) a skeletal visualization of the warning frame. Joint colors represent the relative magnitudes of their contributions to the frame-level $T^{2}$ statistic, ranging from low to high, while labels 1–3 identify the three highest-contributing joints. The table summarizes the corresponding $T^{2}$ values, upper control limits, and monitoring decisions.

Figure 3 presents representative examples of video frames corresponding to low-motion and warning conditions identified by the proposed framework. The warning frame exceeds the Hotelling’s $T^{2}$ upper control limit and corresponds to a period of increased joint motion relative to the task-specific motion distribution. These examples provide a visual illustration of how the statistical monitoring framework identifies motion deviations during task execution.

### 5.2. Benchmarking with Body-Part Ablation

Table 6 presents the mean and standard deviation for the RMSD, mean motion, standard motion, and warning counts across all datasets and ablation types, which was averaged over step sizes and tasks.

Across all datasets, the full body ablation consistently showed the highest RMSD and motion metrics. In the Assembly dataset, full body yielded an RMSD of 0.670 ± 0.313 and a mean motion of 0.829 ± 0.462, while hands and lower body subsets resulted in notably lower values. A similar pattern was observed in G-AI-HMS, where the full body RMSD was 0.761 ± 0.291 compared to 0.229 ± 0.061 for the hands subset.

In the PE-USGC (Near-duplicate) dataset, full body values were substantially higher (RMSD: 3.008 ± 1.598; mean motion: 4.126 ± 1.740) than the partial ablations. UCF Sports Action showed elevated motion values (e.g., full body mean motion: 4.479 ± 2.727) but low warning counts across all ablations. Notably, UCF50 recorded the highest overall RMSD and motion values with full body RMSD reaching 4.184 ± 3.098 and mean motion at 6.942 ± 4.303. However, warning values remained low across datasets except for Assembly, which had the highest warnings under full body (195.292 ± 64.307).

Partial ablations (hands, lower body, upper body) consistently reduced all motion metrics and warnings relative to full body inputs. This trend was stable across the five datasets with low variation in standard deviations between corresponding types of ablation.

Table 7 reports classification performance across five datasets using adaptive thresholding for warning detection. Performance is evaluated using recall, accuracy, precision, and F1-score.

The model trained on the Assembly dataset achieved the highest and most balanced performance across all metrics with recall, accuracy, precision, and F1-score all equal to 0.94, as shown in Table 7. These results indicate strong performance within the source domain. The similar precision and recall values indicate balanced detection performance for the two classes.

For the G-AI-HMS dataset, the model retained performance with scores around 0.80 across all metrics. The slightly higher precision (0.81) compared to recall (0.80) implies that the model was conservative in issuing warnings, successfully avoiding many false alarms while still capturing most true warning instances. Performance decreased on the PE-USGC dataset, with recall dropping to 0.66 and F1-score to 0.63, despite relatively high precision (0.74). This shows that the model accurately identifies warnings when it predicts them but misses many actual warning cases, suggesting under-detection due to domain differences from the Assembly training data.

In the UCF Sports Action dataset, the model achieved a moderate recall of 0.71 but exhibited low precision (0.57) and F1-score (0.53). These results suggest an over-prediction of warnings, which was potentially due to the dynamic nature of sports-related high-risk activity. Similarly, UCF50 produced similar trends with balanced recall and accuracy (0.65) and slightly higher precision (0.68). The F1-score of 0.64 indicates consistent, if modest, classification ability. The large and heterogeneous nature of this dataset likely contributes to its stable but non-optimal performance.

Adaptive thresholding showed the strongest results on datasets structurally similar to the training source (Assembly and G-AI-HMS). Performance dropped on more diverse or high-motion datasets, highlighting the difficulty of using fixed model settings across physical tasks.

## 6. Discussion

The lower camera clarity observed in some S-task recordings may have reduced the accuracy with which the CV algorithm detected subtle postural changes. The perceived motion-demand score on the 0–100 scale was higher for L tasks than for S tasks with mean values of 69.9 and 21.9, respectively, which is consistent with the broader movement trajectories observed during the L tasks. The LGI landmarks (11, 12, 13, 14, 25, and 26) also showed greater spatial deviation than the SGI landmarks (13–22). This observation is consistent with Zhou et al. [53], who used CV to assess student posture and highlighted the influence of camera resolution on detection accuracy. The use of three camera models in this paper was intended to examine the framework under heterogeneous imaging conditions. Real workplace videos may be collected using devices that differ in resolution, optics, compression, and frame rate, and these differences can influence landmark-estimation stability. The results suggest that the proposed motion-monitoring procedure can operate under moderate visual variation, although device-specific performance was not evaluated independently. More severe conditions, including low illumination, substantial compression, and prolonged occlusion, remain important considerations for future evaluation.

The S- and L-task trajectories reflect different underlying movement requirements. The S tasks primarily involved repeated hand positioning, object stabilization, and fine corrections of the wrists, elbows, and fingers. These trajectories were comparatively localized but contained frequent small adjustments. The L tasks required broader arm reaches, trunk movement, bending, and coordination of the upper and lower body. Consequently, variation in the trajectories may reflect differences in task-execution strategy and may indicate motion patterns that warrant further ergonomic review. These observations agree with Ding et al. [54], who developed a real-time webcam-based approach for monitoring upper-body posture and similarly emphasized the relevance of postural variability to ergonomic assessment.

The velocity, motion-amount, and acceleration results indicate that the selected temporal interval affects the sensitivity of the derived motion measures. Consecutive-frame comparisons preserve rapid fluctuations, fine movements, and frame-to-frame landmark variation, whereas Steps 2 and 4 emphasize changes occurring over wider temporal intervals. The lower variability observed at the larger intervals therefore reflects reduced sensitivity to short-duration fluctuations rather than necessarily indicating smoother or safer movement. This interpretation is consistent with Chen et al. [55], who discussed how task complexity can contribute to variability in human performance and behavior.

**Figure 4:**
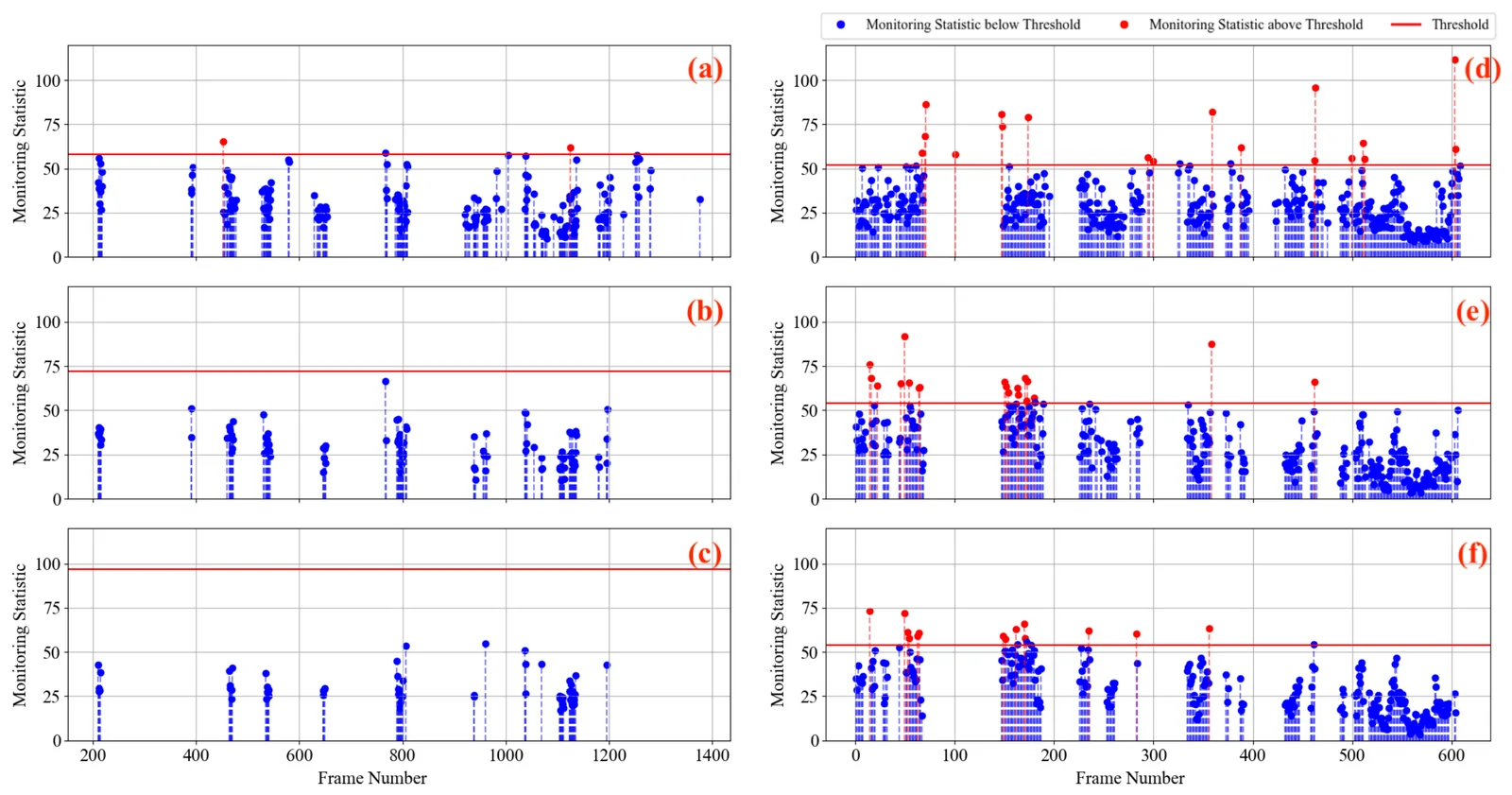
Control charts for SGI: (**a**) Step 0, (**b**) Step 2, (**c**) Step 4 and SGP: (**d**) Step 0, (**e**) Step 2, (**f**) Step 4.

**Figure 5:**
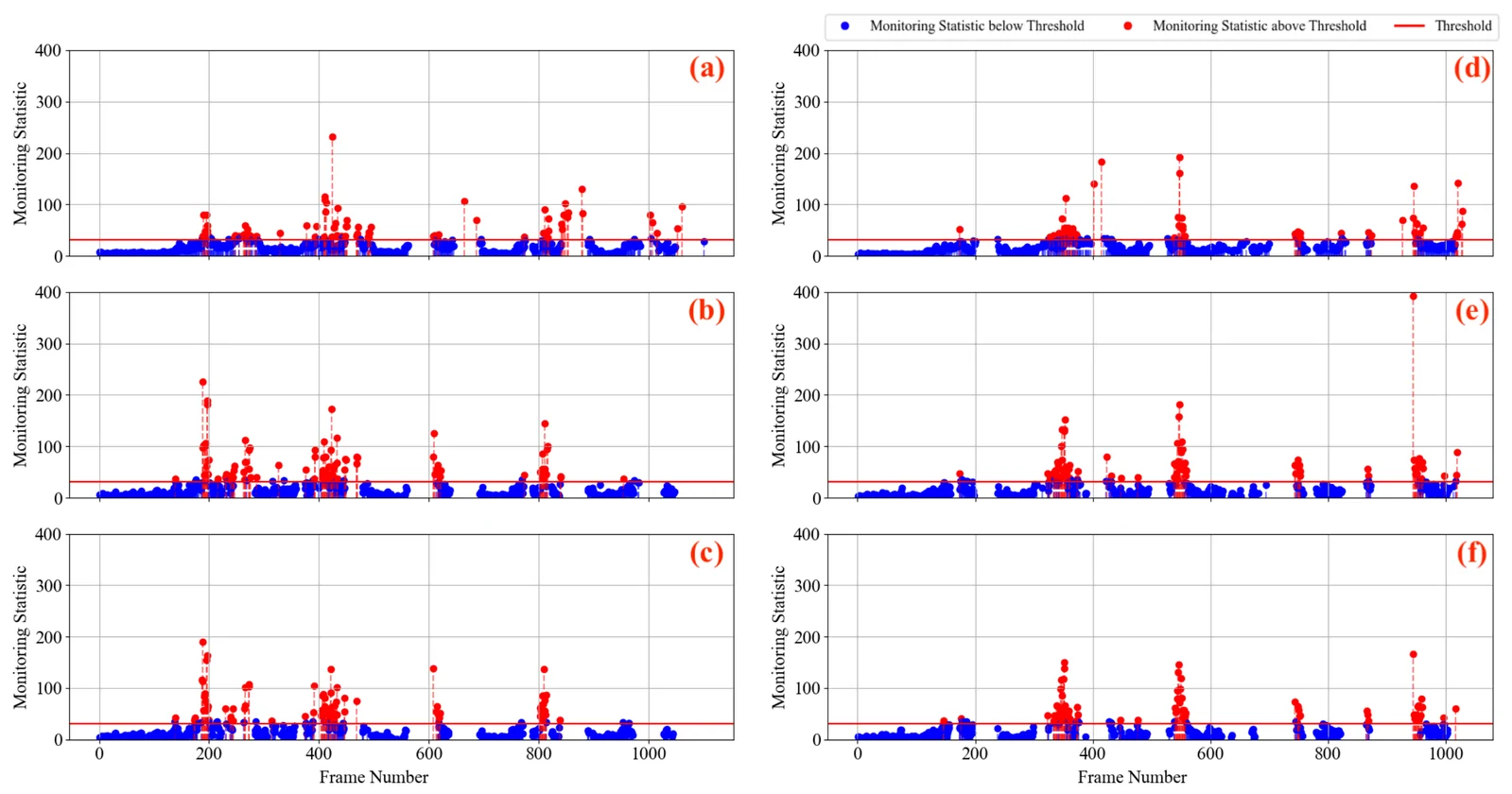
Control charts for LGI: (**a**) Step 0, (**b**) Step 2, (**c**) Step 4 and LGP: (**d**) Step 0, (**e**) Step 2, (**f**) Step 4.

As shown in Figure 4, the small-object tasks produced higher mean $T^{2}$ values than the large-object tasks. A likely mechanism is that the S tasks required sustained fine-motor adjustments during grasping, positioning, and stabilization. Although each adjustment was relatively small, repeated coordinated changes across the hand, wrist, and elbow landmarks produced frequent multivariate deviations from the task-specific mean motion profile, thereby increasing the average $T^{2}$ value. In contrast, the large-object tasks shown in Figure 5 contained broader reaching, lifting, bending, and object-transfer movements. These movements were less continuously variable but occasionally generated substantial departures from the estimated covariance structure, resulting in more extreme $T^{2}$ peaks and warning events. Thus, the higher mean values for the S tasks may represent persistent fine-scale variation, whereas the larger peaks for the L tasks may represent intermittent, higher-magnitude whole-body excursions. The warnings indicate statistical deviations from the observed motion distribution and should not be interpreted as direct evidence of unsafe movement or musculoskeletal injury risk. This interpretation is consistent with research associating task complexity with increased variation and employee strain [56].

The landmark statistics reported in Table 4 further distinguish the spatial demands of the tasks. Small guided tasks, including SGP and SGI, remained concentrated within a relatively limited region of the workspace, reflecting the localized positioning needed to manipulate the cubes. In contrast, the large tasks, including LGI and LGP, showed a wider spatial spread because handling the boxes required longer reaches and greater displacement of the shoulders, elbows, and knees. The larger coordinate variability observed for unguided L tasks may also reflect greater freedom in movement planning and differences in the order and trajectory of object transfer. Mechanistically, guidance constrains the available movement strategies, whereas unguided execution permits greater variation in reach direction, body position, and task sequence. These findings can inform workspace design by supporting closer object placement for precise tasks and providing additional space or mechanical assistance for tasks involving larger objects. This interpretation is consistent with Liu and Li [57], who emphasized the relationship between task complexity, performance, and workspace demands. Compared with observational methods such as REBA [58] and RULA [59], the proposed CV-based framework supports continuous motion tracking and the generation of statistical alerts throughout task execution, which is consistent with prior work on the real-time monitoring of repetitive activities [60].

The cross-dataset classification results reported in Table 7 indicate that performance was influenced by the degree of similarity between each external dataset and the Assembly training data. The Assembly videos contain structured and repeated tasks recorded under relatively controlled viewpoints and environmental conditions, producing aggregate motion-feature distributions that are closely aligned with those used during training. G-AI-HMS [19] also contains comparatively structured motion sequences, which may account for its stronger transfer performance. PE-USGC [12] contains repetitive culinary movements but differs in task scale, motion timing, and camera configuration. UCF Sports Action [20,21] and UCF50 [22] contain faster whole-body movements, greater action diversity, uncontrolled viewpoints, background variation, and more frequent occlusion. These factors can reduce landmark-estimation stability and shift the distributions of RMSD, mean motion, and UCL relative to the Assembly training data. In addition, the adaptive median threshold defines relatively high- and low-warning groups separately within each dataset, meaning that the resulting classification boundary is dataset-dependent rather than an absolute ergonomic threshold. The observed cross-dataset differences therefore reflect combined effects of task-domain shift, visual conditions, motion scale, pose-estimation variability, and relative label construction.

## 7. Conclusions and Future Work

The findings demonstrate the feasibility of the proposed framework under the controlled laboratory conditions evaluated in this paper [61]. In the present implementation, the mean motion vector, covariance matrix, and control limits were estimated using the complete set of frames within each analyzed task segment. Therefore, the reported warning identification represents retrospective Phase I monitoring rather than prospective real-time detection. A prospective real-time implementation would require establishing the reference motion distribution and control limits using previously collected baseline task data and subsequently applying these fixed, or adaptively updated, parameters to incoming video frames. Such a prospective real-time evaluation was beyond the scope of this paper. However, given the subjective and multifactorial nature of NASA-TLX, this modest correlation should be interpreted as complementary rather than confirmatory ergonomic evidence. By analyzing tasks, professionals can identify motion patterns that may warrant further ergonomic review and develop workplace-specific interventions. For example, if the framework detects frequent reaching movements through z-coordinate analysis [62], an ergonomist may review the task to determine whether mechanical aids or workspace adjustments are appropriate. Simulating task and layout changes in virtual environments offers ergonomic insights before real-world modifications are made [63]. Long-term motion tracking enables monitoring the effectiveness of ergonomic solutions, facilitating the continuous assessment of workspace changes and strategy adjustments. Motion analysis also helps predict and prevent future issues by minimizing estimation errors [64]. Within the controlled experimental setting, the framework detected motion variations that showed a modest association with perceived task demand. These findings should not be generalized to broader worker populations or uncontrolled industrial environments. The framework provides statistical motion alerts intended to support preliminary ergonomic screening. These alerts indicate deviations from the observed motion distribution and should not be interpreted as a clinical diagnosis or definitive assessment of musculoskeletal injury risk. However, this paper has several limitations. First, the analysis involved only two male participants with similar anthropometric characteristics, which limits the generalizability of the findings across diverse populations, genders, and body types. The limited sample size should not be interpreted as a validation of population-level performance. Rather, the collected dataset was intended to support methodological validation of the proposed framework under controlled experimental conditions. Future studies should evaluate the framework using larger and more diverse participant cohorts to better assess its generalizability across anthropometric characteristics, genders, occupations, and movement patterns. Second, the videos were recorded from fixed, laboratory-based camera angles under controlled lighting conditions; therefore, the framework’s performance in dynamic or cluttered industrial environments remains to be evaluated. Third, the system depends on the accuracy of posture estimation and was not directly validated against a gold-standard optical motion-capture system within this paper. A complementary study by the authors validated body motion estimation using synchronized EMG and IMU measurements across multiple occupational task domains, providing independent evidence of the relationship between physiological muscle activation and body movement [65]. Nevertheless, future work will include direct validation against optical motion-capture systems to further quantify the accuracy of the proposed computer vision framework. Fourth, the Random Forest classifier was selected as a representative supervised learning model to evaluate the proposed framework and was not intended as a comparison of machine learning algorithms. Future work should investigate alternative classification methods to determine whether additional performance gains can be achieved across diverse task domains. Finally, classification performance was evaluated using labels derived from the proposed Hotelling’s $T^{2}$-based monitoring framework rather than independent ergonomic risk annotations. Validation against expert ergonomic assessments and larger participant cohorts will further strengthen the applicability of the proposed approach. The classification performance reported in this paper was evaluated against labels derived from the Hotelling’s $T^{2}$ monitoring framework rather than independently annotated ergonomic risk assessments. Consequently, the reported classification accuracy reflects agreement with the proposed statistical monitoring approach and should not be interpreted as validation against a clinical or ergonomic ground-truth standard. Future work will include external validation using expert ergonomic assessments and established biomechanical measurement systems.

Future work will address these limitations by (i) conducting larger-scale, multi-participant studies across genders and occupations, (ii) incorporating adaptive camera calibration and occlusion-handling techniques, and (iii) integrating synchronized IMU/EMG wearable data for cross-validation [65]. Future research will focus on designing scalable human subject experiments to further evaluate CV-based methods. In addition, although the proposed framework is designed for real-time deployment, this paper did not include a formal evaluation of processing time, end-to-end latency, or achievable frame rate. Future work will include computational benchmarking under different hardware configurations to characterize real-time performance. In conclusion, this paper serves as a methodological pilot evaluation of a CV-based framework for motion representation under controlled laboratory conditions. Although the proposed framework demonstrated promising performance across the collected and benchmark datasets, additional validation using larger and more diverse participant populations, gold-standard motion measurement systems, and real-world industrial environments is needed before broader deployment. The full implementation code and supporting datasets are publicly available on GitHub [66].

## Figures and Tables

**Table 1:** Notation used in motion quantification and risk detection framework.

Symbol	Definition	Dimensions
$v_{i,k}$	3D position of landmark *i* at frame *k*	$R^{3}$
$m_{i,k}$	Motion vector of landmark *i* in frames	$R^{3}$
$m_{k}$	Concatenated motion vector *k*	$R^{3p}$
$M_{k}$	Total motion magnitude in frame *k*	Unitless
$\bar{M}$	Mean motion across all frames	Unitless
${\mathrm{RMSD}}_{\mathrm{task}}$	RMSD for task segment	Unitless
V	Position matrix for landmarks in frames	K×3p matrix
S	Covariance matrix of motion vectors	$R^{3p\times 3p}$
$t_{k}$	Hotelling’s $T^{2}$ statistic for frame *k*	Unitless
T	Vector of $T^{2}$ values across frames	$R^{K}$
UCL	Upper control limit from F-distribution	Unitless
LCL	Lower control limit (typically 0)	Unitless
$y_{i}$	True warning label for segment *i*	{0, 1}
${\hat{y}}_{i}$	Predicted warning label for segment *i*	{0, 1}
$\tau_{d}$	Median warning threshold for dataset *d*	Integer (count)
$x_{i}$	Feature vector: RMSD, mean motion, UCL	$R^{3}$
$f_{\theta }$	Trained Random Forest classifier	$R^{3}\to \left\{0,1\right\}$
$h_{j}$	Decision tree *j* in the forest	$R^{3}\to \left\{0,1\right\}$
$\mu_{\mathrm{motion}}$	Mean joint motion for a segment	Unitless
$\sigma_{\mathrm{motion}}$	Standard deviation of joint motion	Unitless

**Table 2:** Landmark points for S and L tasks.

Index	Body Part	Task Type
11	Left shoulder	L
12	Right shoulder	L
13	Left elbow	S and L
14	Right elbow	S and L
15	Left wrist	S
16	Right wrist	S
17	Left pinky	S
18	Right pinky	S
19	Left index	S
20	Right index	S
21	Left thumb	S
22	Right thumb	S
25	Left knee	L
26	Right knee	L

**Table 3:** Statistics for joint kinematics at different frame intervals. Motion amount is dimensionless, velocity is reported in normalized-coordinate units per frame, and acceleration is reported in normalized-coordinate units per frame squared.

Data Type	Statistic	Step 0	Step 2	Step 4
Motion Amount	Count	1099	1097	1095
	Mean	0.097	0.052	0.057
	SD	0.086	0.068	0.072
Velocity	Count	1099	1097	1095
	Mean	0.012	0.003	0.002
	SD	0.010	0.004	0.002
Acceleration	Count	1098	1096	1094
	Mean	0.0006	0.00004	−0.00001
	SD	0.311	0.078	0.02

**Table 4:** Statistics for landmark point movement in normalized coordinate space; all reported coordinate values are dimensionless.

Task	Mean			SD		
	X	Y	Z	X	Y	Z
SGI	0.604	0.546	0.011	0.018	0.021	0.035
SGP	0.611	0.527	0.023	0.011	0.020	0.047
SUI	0.609	0.523	0.015	0.012	0.022	0.050
SUP	0.607	0.530	0.020	0.013	0.019	0.045
LGI	0.534	0.378	−0.134	0.080	0.055	0.140
LGP	0.537	0.385	−0.120	0.085	0.065	0.150
LUI	0.532	0.370	−0.125	0.075	0.050	0.130
LUP	0.529	0.365	−0.115	0.070	0.045	0.120

**Table 5:** Correlation between motion warnings and NASA-TLX subscales.

NASA-TLX Subscale	Correlation (r)	*p*-Value
Mental Demand	0.192	p<0.01
Physical Demand	0.214	p<0.005
Temporal Demand	0.231	p<0.005
Performance	0.109	p=0.13
Effort	0.155	p<0.05
Frustration	—	—
**Average**	**0.218**	p<0.005

**Table 6:** Descriptive statistics by dataset and ablation type averaged over step sizes and tasks. RMSD, motion mean, and motion standard deviation are dimensionless, while warnings are reported as counts.

Dataset	Ablation	RMSD	Motion Mean	Warnings
G-AI-HMS [19]	Full Body	0.761 ± 0.291	0.950 ± 0.297	103.375 ± 114.671
	Hands	0.229 ± 0.061	0.297 ± 0.107	105.333 ± 91.716
	Lower Body	0.307 ± 0.152	0.304 ± 0.118	107.208 ± 101.138
	Upper Body	0.293 ± 0.076	0.386 ± 0.128	109.875 ± 103.227
PE-USGC [12]	Full Body	3.008 ± 1.598	4.126 ± 1.740	114.958 ± 90.720
	Hands	0.667 ± 0.357	0.864 ± 0.469	108.991 ± 64.320
	Lower Body	1.189 ± 0.570	1.678 ± 0.608	113.852 ± 69.345
	Upper Body	0.951 ± 0.513	1.231 ± 0.648	113.620 ± 71.310
UCF Sports Action [20,21]	Full Body	2.434 ± 1.656	4.479 ± 2.727	0.000 ± 0.000
	Hands	0.757 ± 0.435	1.221 ± 0.727	0.766 ± 2.794
	Lower Body	0.767 ± 0.496	1.368 ± 0.913	0.286 ± 1.362
	Upper Body	1.013 ± 0.624	1.658 ± 0.988	0.234 ± 1.366
UCF50 [22]	Full Body	4.184 ± 3.098	6.942 ± 4.303	14.743 ± 54.658
	Hands	0.962 ± 0.708	1.408 ± 0.976	13.122 ± 47.737
	Lower Body	1.289 ± 1.010	2.029 ± 1.487	13.923 ± 49.991
	Upper Body	1.604 ± 1.200	2.473 ± 1.761	13.672 ± 48.764
Assembly (current study)	Full Body	0.670 ± 0.313	0.829 ± 0.462	195.292 ± 64.307
	Hands	0.252 ± 0.103	0.250 ± 0.119	154.833 ± 41.731
	Lower Body	0.210 ± 0.165	0.233 ± 0.181	161.500 ± 42.953
	Upper Body	0.315 ± 0.127	0.331 ± 0.161	174.146 ± 45.950

**Table 7:** Classification performance (recall, accuracy, precision, and F1-score) using adaptive thresholding.

Dataset	Recall	Acc.	Precision	F1
G-AI-HMS [19]	0.80	0.80	0.81	0.80
PE-USGC [12]	0.66	0.66	0.74	0.63
UCF Sports Action [20,21]	0.71	0.65	0.57	0.53
UCF50 [22]	0.65	0.65	0.68	0.64
Assembly (current study)	0.94	0.94	0.94	0.94

## Data Availability

The data supporting the findings of this paper are publicly available in the data/ folder of the project’s GitHub repository [66].

## Abbreviations

The following abbreviations are used in this manuscript:

3D	Three-dimensional
AI	Artificial intelligence
CSV	Comma-separated values
CV	Computer vision
EMG	Electromyography
F1	F1-score
FN	False negative
FP	False positive
G-AI-HMS	Generative-AI-Enabled Human Motion Simulation
GPU	Graphics processing unit
HMS	Human motion simulation
ID	Identifier
IMU	Inertial measurement unit
IRB	Institutional Review Board
LCL	Lower control limit
LGI	Large guided insertion task
LGP	Large guided placement task
LUI	Large unguided insertion task
LUP	Large unguided placement task
MVFNet	Multi-View Fusion Network
PE-USGC	Posture Estimation-Based Unsupervised Spatial Gaussian Clustering
REBA	Rapid Entire Body Assessment
RGB	Red, green, and blue
RMSD	Root mean square deviation
RULA	Rapid Upper Limb Assessment
SD	Standard deviation
SGI	Small guided insertion task
SGP	Small guided placement task
SUI	Small unguided insertion task
SUP	Small unguided placement task
SVM	Support vector machine
TLX	Task Load Index
TN	True negative
TP	True positive
UCF	University of Central Florida
UCF50	UCF50 Action Recognition Dataset
UCL	Upper control limit
